# Dr. Darwall

**Published:** 1833-10

**Authors:** 


### OBITUARY.

DIED, on the 10th of August, at Birmingham, Dr. DARWALL. His death was occasioned by an injury received in the examination of a dead body, at the Birmingham Hospital, on the 30th of July. He was a man of the most ardent and enlightened benevolence, and the most indefatigable industry, and united a profound knowledge of medicine to great practical skill. His premature decease (for he was not yet forty years of age) is a loss, not only to his friends, but to society.

